# Assessing cognition in autistic youth with and without attention‐deficit/hyperactivity disorder using the NIH Toolbox Cognition Battery: An Environmental influences on Child Health Outcomes‐Wide Cohort Study

**DOI:** 10.1002/jcv2.70031

**Published:** 2025-07-16

**Authors:** Catrina A. Calub, Aisha S. Dickerson, Haozuo Zhao, Robert M. Joseph, Michael O'Shea, Shaikh I. Ahmad, Lisa A. Croen, Sean C. L. Deoni, Viren A. D’Sa, Akhgar Ghassabian, Daphne Koinis‐Mitchell, Leonardo Trasande, Heather Volk, Anna J. Yeo, Irva Hertz‐Picciotto, Julie B. Schweitzer, Marjorie Solomon, for the ECHO Cohort Consortium

**Affiliations:** ^1^ Department of Psychiatry and Behavioral Sciences University of California, Davis Sacramento California USA; ^2^ MIND Institute University of California, Davis Sacramento California USA; ^3^ Department of Epidemiology Johns Hopkins Bloomberg School of Public Health Baltimore Maryland USA; ^4^ Boston University Chobanian & Avedisian School of Medicine Boston Massachusetts USA; ^5^ Department of Pediatrics University of North Carolina at Chapel Hill Chapel Hill North Carolina USA; ^6^ University of California, San Francisco San Francisco California USA; ^7^ Division of Research Kaiser Permanente Northern California Pleasanton California USA; ^8^ Maternal, Newborn, and Child Health Discovery & Tools Bill & Melinda Gates Foundation Seattle Washington USA; ^9^ Rhode Island Hospital Warren Alpert Medical School of Brown University Providence Rhode Island USA; ^10^ Department of Pediatrics NYU Grossman School of Medicine New York New York USA; ^11^ Department of Pediatrics Hasbro Children's Hospital/Alpert Medical School of Brown University Providence Rhode Island USA; ^12^ Department of Psychiatry and Human Behavior Alpert Medical School of Brown University Providence Rhode Island USA; ^13^ Department of Population Health NYU Grossman School of Medicine New York New York USA; ^14^ Department of Public Health Sciences University of California, Davis Davis California USA

**Keywords:** adolescents, attention‐deficit/hyperactivity disorder (ADHD), autism spectrum disorder, cognitive functioning, ECHO, latent profile analysis, NIH Toolbox

## Abstract

**Background:**

Prior work has suggested poorer performance on Fluid, but not Crystallized, NIH Toolbox Cognition Battery (NTCB) subtests in autistic youth relative to neurotypical individuals. This study sought to extend previous findings using a larger sample from a nationwide multi‐cohort study and to examine the effect of attention‐deficit/hyperactivity disorder (ADHD) status on cognitive functioning.

**Methods:**

We used NTCB data from 1035 children ages 12–18 in five cohorts drawn from the Environmental influences on Child Health Outcomes (ECHO) Program. Participants with autism only (AUT; *n* = 81), ADHD only (*n* = 162), co‐occurring autism and ADHD (AUT + ADHD; *n* = 55), and a comparison group with neither autism nor ADHD (noAUTADHD; *n* = 737) were included in the analyses. We used a general linear model framework to examine group differences in task performance, and we used latent profile analysis (LPA) to identify subgroups of individuals with similar cognitive profiles.

**Results:**

The AUT and AUT + ADHD groups had lower NTCB Fluid Cognition Index Scores compared to the noAUTADHD group, whereas no group differences in the Crystallized Cognition Index Score were observed. No significant differences in the NTCB subtest or index scores were found between the AUT and AUT + ADHD groups. Sex‐stratified analyses revealed no sex differences. LPA identified four distinct NTCB groups, with a substantial proportion of the AUT group (19%) assigned to the class with higher Crystallized versus Fluid Cognition Index Scores; however, there was considerable overlap between the diagnostic groups within the LPA classes.

**Conclusions:**

Autistic individuals experience difficulties with fluid NTCB tasks and those with co‐occurring ADHD do not appear to face greater difficulties than those with autism alone. However, there was a notable proportion of autistic individuals with average or superior cognitive performance, highlighting the importance of strength‐based and transdiagnostic research approaches.

## Key Points

- Crystallized cognition may be an area of relative strength on the NIH Toolbox Cognition Battery (NTCB), and fluid cognition an area of relative weakness, for autistic youth with and without attention‐deficit/hyperactivity disorder (ADHD).
- Autistic youth with and without ADHD perform similarly on the NTCB, suggesting ADHD may have minimal impact on their performance on cognitive measures.
- Participants, both with and without autism or ADHD, were classified into each of the latent profile analysis groups, suggesting transdiagnostic overlap in their cognitive profiles.
- Further investigation to better understand the heterogenity of cognitive profiles of autistic individuals, including cognitive strengths and challenges, is warranted as it may inform the development of targeted interventions.

## Graphical abstract

Autistic individuals tend to experience difficulties with fluid cognition as found previously, with minimal impact of ADHD. However, there was a notable proportion of autistic individuals with average or superior cognitive performance, highlighting the importance of strength‐based and transdiagnostic research approaches.

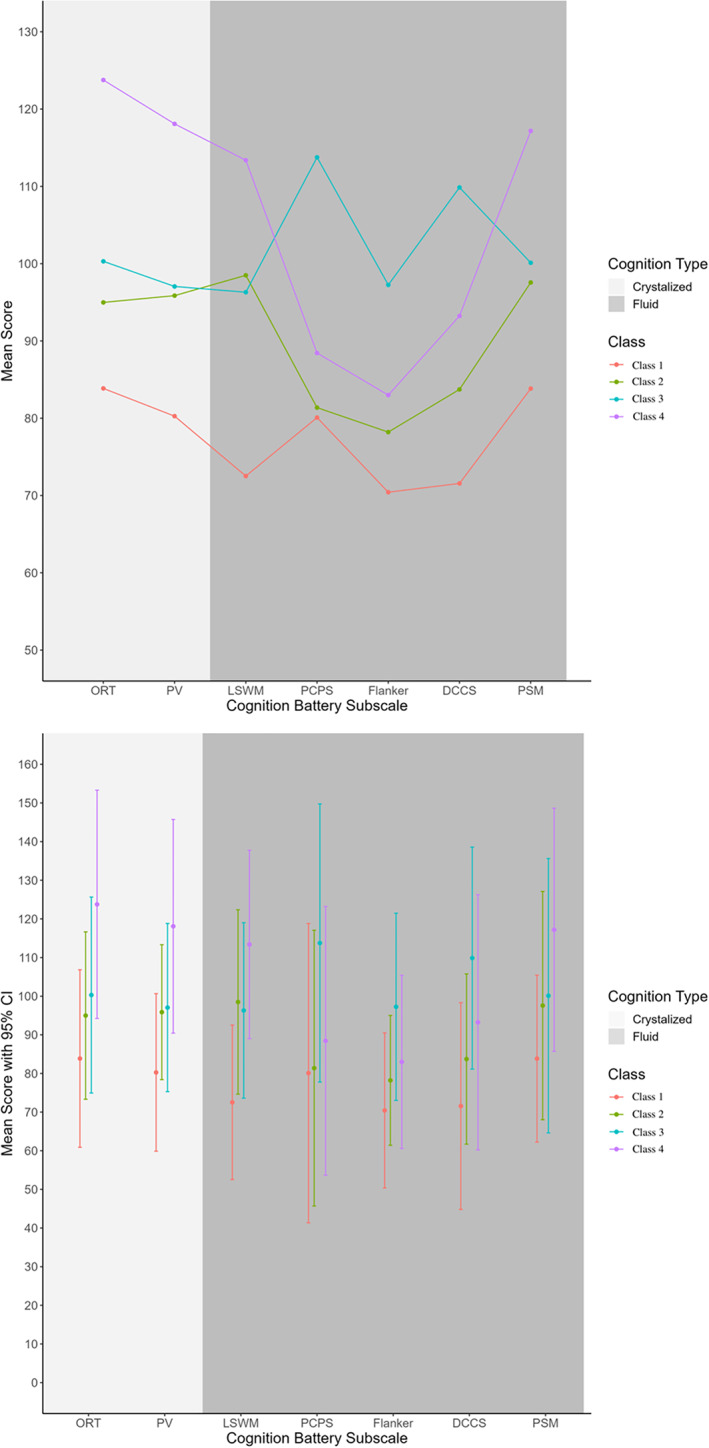

## INTRODUCTION

Autism is a neurodevelopmental disorder that is characterized by difficulties with social communication and interaction, and restricted and repetitive behaviors, interests, and activities (American Psychological Association, 2013). Additionally, autism is associated with cognitive challenges that are in turn associated with poorer academic performance (Ameis et al., 2022), social skills (Leung et al., 2016), adaptive functioning (Kenny et al., 2019), response to treatment (Antshel et al., 2011; Calub et al., 2022), and overall quality of life (de Vries & Geurts, 2015). Thus, a better understanding of the cognitive profiles of autistic individuals, including their cognitive strengths and challenges, is clinically relevant and may inform the development of targeted interventions (Charman et al., 2011).

Solomon et al. (2021) examined the cognitive profiles of an intelligence quotient (IQ) case‐matched cohort with autism (*n* = 66) and neurotypical (*n* = 66) individuals ages 12–22 (Solomon et al., 2021) using latent profile analysis (LPA) and the National Institutes of Health (NIH) Toolbox Cognition Battery (NTCB). While only reflecting one model of cognitive processes, the NTCB is a widely used cognitive assessment tool that yields two scores that are statistical composites intended to reflect aspects of fluid and crystallized cognition (Weintraub et al., 2013). The Fluid Composite Index includes subtests intended to measure response inhibition, working memory, set‐shifting, episodic memory, and processing speed, whereas the Crystallized Composite Index includes subtests measuring vocabulary and oral reading. While fluid abilities are associated with solving novel problems that tends to decline with age, crystallized abilities involve applying knowledge acquired through experience and education to solve problems, which often improves or remains stable over time (Schneider & McGrew, 2018). The LPA conducted by Solomon et al. (2021) identified four subgroups of cognitive profiles that differed in their membership of autistic and neurotypical individuals. Most autistic individuals belonged to either the class with higher scores on the Crystallized subtests compared with the Fluid subtests or the class with low scores overall. Additionally, they showed that the autistic group performed more poorly than the neurotypical group in Fluid, but not Crystallized, subtests. Importantly, their results revealed that a subset of autistic participants performed comparably to their neurotypical peers, suggesting that not all autistic persons have the same weaknesses, and that cognitive profiles may overlap between those with and without autism. Interestingly, no significant correlation was observed between NTCB subtest scores and parent‐reported attention‐deficit/hyperactivity disorder (ADHD) symptoms, which may have been due to the relatively small sample. Examining cognition in autistic individuals with comorbid ADHD warrants further investigation considering the high prevalence of ADHD diagnoses among autistic individuals (Rong et al., 2021) and evidence that ADHD symptoms significantly impact overall functioning (Rosello et al., 2022).

The primary purpose of this study was to assess the replicability of Solomon et al. (2021) using a larger, more diverse, and community‐based sample. This aligns with the growing calls in the scientific community to prioritize replication as means of ensuring that findings are valid, reliable, and not artifacts of sample or context (e.g., Buck, 2024; Editorial staff of Nature Human Behavior, 2024). We included groups with autism only (AUT), ADHD only (ADHD), comorbid autism and ADHD (AUT + ADHD), and individuals without autism or ADHD (NoAUTADHD). We leveraged data from five cohorts in the nationwide multi‐site Environmental influences on Child Health Outcomes (ECHO) Program funded by the NIH. Within this large sample, we aimed to further examine the effects of ADHD status on cognitive functioning in autistic youth using the NTCB. We also aimed to examine whether different diagnostic groups (AUT, ADHD, NoAUTADHD) have characteristic cognitive profiles using LPA. We hypothesized that individuals with autism and/or ADHD would perform poorer in the Fluid, but not Crystallized, subtests than their peers without ADHD or autism, consistent with previous findings (Solomon et al., 2021). We further hypothesized that autistic youth without ADHD would perform better in the Fluid subtests than autistic youth with ADHD (Rosello et al., 2022). Lastly, we hypothesized that most autistic youth would belong to a class with all low scores or a class with lower Fluid cognition scores than Crystallized cognition scores and that a subset of autistic individuals would perform comparably to their neurotypical peers (Solomon et al., 2021).

## METHODS

### Study population

ECHO is a multi‐cohort consortium that was established to disentangle environmental factors that impact various aspects of child health. In summary, in its first phase (Cycle 1), ECHO consisted of 69 cohorts that utilized a common protocol for assessing environmental exposures and various child health outcomes, including birth outcomes, respiratory health, obesity, overall wellbeing, and neurodevelopment (Gillman & Blaisdell, 2018). For this analysis, we included only children who had NTCB assessment scores (*n* = 11,701). We then excluded any child participants missing the Fluid or Crystalized Composite Indices of the NTCB (*n* = 6491), those who were not 12–18 years of age at the time the NTCB was administered (*n* = 3266), those missing total NIH Composite Scores or any subtest score (*n* = 25), those missing parent‐reported autism or ADHD status information (*n* = 271), and those missing an IQ score or with an IQ score <70 (*n* = 613). This allowed for the analysis of data from 1035 children enrolled in five ECHO cohorts with a mean ± standard deviation (SD) age of 15 ± 2 years and an IQ score of 101 ± 13.3 (Figure S1 and Table 1). The study protocol was approved by the single ECHO Institutional Review Board, WCG IRB. Written informed consent or parent's/guardian's permission was obtained along with child assent as appropriate, for the ECHO Cohort Data and Biospecimen Collection Protocol participation and for participation in specific study sites.

**TABLE 1:** Sociodemographic characteristics of the participating children.

	NoAUTADHD	AUT	ADHD	AUT + ADHD	Total sample	*p‐*value
	(*n* = 737)	(*n* = 81)	(*n* = 162)	(*n* = 55)	(*n* = 1035)	
Sex, *n* (%)						
Male	347 (47.1%)	61 (75.3%)	106 (65.4%)	41 (74.5%)	555 (53.6%)	<0.001
Female	390 (52.9%)	20 (24.7%)	56 (34.6%)	14 (25.5%)	480 (46.4%)	
Race						
White	410 (55.6%)	60 (74.1%)	109 (67.3%)	42 (76.4%)	621 (60.0%)	<0.001
Black	250 (33.9%)	7 (8.6%)	36 (22.2%)	8 (14.5%)	301 (29.1%)	
Other/multiple race	70 (9.5%)	13 (16.0%)	14 (8.6%)	5 (9.1%)	102 (9.9%)	
Missing	7 (0.9%)	1 (1.2%)	3 (1.9%)	0 (0%)	11 (1.1%)	
Ethnicity						
Hispanic	64 (8.7%)	18 (22.2%)	9 (5.6%)	7 (12.7%)	98 (9.5%)	<0.001
Non‐Hispanic	673 (91.3%)	63 (77.8%)	153 (94.4%)	48 (87.3%)	937 (90.5%)	
Age (years), mean (SD)	15.0 (2.0)	15.1 (2.1)	15.1 (1.8)	15.4 (2.0)	15.0 (2.0)	0.437
IQ score, mean (SD)	102.3 (13.5)	95.3 (13.8)	100.7 (14.6)	98.6 (14.6)	101 (13.9)	0.0241

*Note*: *p‐*values for differences between diagnostic groups are based on Chi‐squared tests of significance for sex, race, and ethnicity, and one‐way analysis of variance (ANOVA) for age and IQ scores.

Abbreviations: ADHD, attention deficit hyperactivity disorder; AUT, autism spectrum disorder; AUT + ADHD, co‐occurring autism and ADHD; IQ, intelligence quotient; NoAUTADHD, no autism or ADHD; SD, standard deviation.

### Neurodevelopmental outcomes

ADHD and autism diagnostic status were based on parent or caregiver report of a clinician diagnosis based on diagnostic questionnaires, medical history, and demographic information forms. Children were categorized into the NoAUTADHD group if they had no parent‐reported diagnosis of autism or ADHD. Individuals missing information on reported autism diagnosis were not included. For children with multiple NTCB assessments, we used the results for the first assessment administered within the specified age range. Information on child sex was obtained across cohorts from questionnaires and medical record abstraction. IQ scores were derived from a variety of standardized measures based on site preference, including the Wechsler Abbreviated Scale of Intelligence‐Second Edition (WASI‐II; Wechsler, 1999), Wechsler Intelligence Scale for Children‐Third and Fifth Editions (WISC‐III, WISC‐V; Na & Burns, 2016), and the Stanford‐Binet Intelligence Scales, Fifth Edition (SB‐5; Roid & Pomplun, 2012). ECHO sites selected their preferred measures, consistent with practices in large multi‐site longitudinal studies. Although data were collected at varying time points, including some archival assessments (range: 8.1–15.4 years), prior research suggests that IQ remains stable across this age range (e.g., Yu et al., 2018). While the IQ measures used across the ECHO cohorts are not fully concordant (e.g., Farmer et al., 2015; Silverman et al., 2011), they are highly intercorrelated and are therefore considered adequate and appropriate for use in the current analyses.

### NIH Toolbox Cognitive Battery

The NTCB is a compilation of seven brief tests typically administered via a computer or tablet (Akshoomoff et al., 2014). The details of each subtest have been described previously (Solomon et al., 2021). The NTCB provides two statistical composite indices: Fluid and Crystallized. It should be noted that NTCB indices are widely used and considered to be a psychometrically valid (Akshoomoff et al., 2014; Zelazo et al., 2013) way to measure *one* conceptualization of cognition. The Fluid Composite Index is intended to reflect a child's ability to problem solve, think quickly, and adapt. The Fluid Index includes the Dimensional Change Card Sort (DCCS) Test to assess set‐shifting/cognitive flexibility (Zelazo, 2006), Flanker Inhibitory Control and Attention Test (Flanker) to assess response inhibition (Eriksen & Eriksen, 1974), List Sorting Working Memory (LSWM) Test to assess working memory (Tulsky et al., 2014), Pattern Comparison Processing Speed (PCPS) Test to assess processing speed (Carlozzi et al., 2015), and Picture Sequence Memory Test to assess episodic memory. The Crystalized Composite Index is intended to reflect vocabulary and oral reading (Akshoomoff et al., 2014) and includes the 25‐item Picture Vocabulary Test of receptive vocabulary and the Oral Reading Recognition Test of literacy, respectively. The NTCB provides two *T*‐score values: uncorrected and age‐ and gender‐corrected. For all analyses with the exception of the sex‐stratified analyses, the age‐ and gender‐corrected *T*‐scores were used.

### Statistical analysis

Similar to Solomon et al. (2021), the present analyses examine diagnostic group differences on the NTCB and employs LPA on an IQ‐matched sample to identify subgroups based on cognitive performance. While Solomon et al. (2021) compares AUT with neurotypical individuals and examines age‐related changes, the present analyses explore four groups: autism alone (AUT), ADHD alone (ADHD), co‐occurring autism and ADHD (AUT + ADHD), and those without autism or ADHD (noAUTADHD), and includes additional analyses examining interactions between autism and ADHD diagnoses and sex‐based stratifications.

For the present study, the descriptive statistics for the study population, including sex, age, and IQ score, were examined by diagnostic status. Specifically, we stratified these analyses based on diagnostic group (AUT, ADHD, AUT + ADHD, NoAUTADHD). Diagnostic group differences in NTCB subtest measures and composite indices were assessed using analysis of variance (ANOVA) tests, adjusting multiple comparisons using a Holm‐Bonferroni correction (Holm, 1979). We also tested for an interaction between the Fluid and Crystallized Composite Indices. When ANOVA results were statistically significant, we used post‐hoc Tukey's honestly significant difference tests to determine which groups were different from others. We used generalized linear models (GLMs) with post‐hoc adjustment for individual cohorts to examine associations between diagnostic groups and Fluid and Crystalized Composite scores. Because there were group differences in IQ, we then added IQ scores to the model and tested for an effect modification via interaction terms. In analyses of the associations between IQ scores and NTCB Composite scores, we also tested for an interaction between autism and ADHD diagnosis and we stratified the analysis by diagnostic group to further examine the effect modification. For these analyses, which were stratified a priori by sex, we used the uncorrected Fluid and Crystalized Composite *T*‐scores and adjusted them for age to facilitate a more focused examination of each gender individually.

We then performed an LPA to examine the profiles of NTCB scores in the sample using an IQ matched sample. Given the cognitive heterogeneity across cohorts—including variability in support needs and demographic composition—IQ matching was necessary to reduce confounding and enhance the interpretability of profile differences in NTCB scores. Therefore, similar to the Solomon et al. study, we 1:1:1 matched each member of the ADHD group to one NoAUTADHD child and one member of the AUT group based on IQ scores using the automated greedy matching algorithm. This matching process ensured minimal differences in IQ scores between those with autism or ADHD and those without these disorders. The IQ‐matched sample consisted of 243 participants, with 81 individuals in each of the AUT, ADHD, and NoAUTADHD groups. Children (NoAUTADHD: *n* = 656, ADHD: *n* = 81, AUT + ADHD: *n* = 55) who could not be matched were excluded from the IQ matched sample to maintain the integrity of the 1:1:1 matching structure. The LPA was constructed using the NTCB subtests as covariates. We compared LPA models divided into 2–5 classes to assess the best fit for the model. Specifically, goodness‐of‐fit was determined using the Akaike information criterion (AIC), Bayesian information criterion (BIC), entropy, and Parametric Bootstrapped likelihood ratio test (PBLRT; Kim, 2014). Smaller AIC and BIC values indicated better fit; an entropy value closer to one indicated better classification; and significant *p*‐values for PBLRT indicated a better fit of each class to the model. Study participants were subsequently assigned to different classes in the model with the best fit according to the posterior probability, and we compared IQ scores, Fluid and Crystalized Composite Indices, sex, and diagnostic groups by these latent classes. We also conducted the LPA with our unmatched sample. Findings are largely similar and can be found in Tables S2 and S3.

Lastly, due to potential differences in neurodevelopmental trajectories for preterm children and those recruited from neonatal intensive care units (NICUs), we conducted sensitivity analyses. Specifically, we re‐ran the ANOVA, GLM, and LPA after excluding the NICU cohort to assess the robustness of our findings. The descriptive statistics for this subsample are displayed in Table S4.

All statistical analyses were performed using SAS, version 9.4, and R, version 4.1.0, with an alpha level of 0.05 adjusted for multiple comparisons with a Holm‐Bonferroni correction.

## RESULTS

### Sociodemographic characteristics

The total sample consisted of 1035 participants across the NoAUTADHD (*n* = 737), AUT (*n* = 81), ADHD (*n* = 162), and AUT + ADHD (*n* = 55) groups. The overall mean age was 15.0 (SD = 2.0) years. There were fewer males (47.1%) in the noAUTADHD group relative to the AUT (75.3%), ADHD (65.4%), and AUT + ADHD (74.5%) groups. The AUT group had a higher proportion of White individuals (76.4%) and a lower proportion of Black individuals (8.6%) compared to the NoAUTADHD group (White: 55.6%, Black: 33.9%) and the ADHD group (White: 67.3%, Black: 22.2%). The AUT group also had a significantly higher proportion of Hispanic individuals (22.2%) compared to the NoAUTADHD group (8.7%) and the ADHD group (5.6%). IQ scores were lower for the AUT group (mean = 95.3, SD = 95.3) compared to noAUTADHD (mean = 102.3, SD = 13.5) and ADHD (mean = 100.7, SD = 14.6). There were no other significant sociodemographic characteristics group differences (see Table 1).

### NIH Toolbox Cognitive Battery

The means and SDs for the NTCB subtest scores are presented in Table 2. The results showed that while the Crystallized Composite Index scores did not differ between diagnostic groups (*p* = 0.17), the Fluid Composite Index scores did differ (*p* < 0.001), with AUT and AUT + ADHD children having lower Fluid Composite Index scores than the NoAUTADHD group (Cohen's *d* = −0.45 and −0.65, respectively). Additionally, the AUT + ADHD group had lower Fluid Composite Index scores than the ADHD group (Cohen's *d* = −0.65). Significant differences were also found between the diagnostic groups for several subscales of the Fluid Composite Index, including the DCCS (*p* < 0.001), LSWM (*p* = 0.002), and PCPS (*p* < 0.001), but no subscales differences were observed for the Crystallized Composite Index. For the DCCS subscale, the AUT group (Cohen's *d* = −0.51) and AUT + ADHD group (Cohen's *d* = −0.69) scored lower than the NoAUTADHD group. On the LSWM subscale, the AUT group scored lower than the NoAUTADHD group (Cohen's *d* = −0.35). Similarly, on the PCPS subscale, the AUT group (Cohen's *d* = −0.42) and AUT + ADHD group (Cohen's *d* = −0.43) scored lower than the NoAUTADHD group. No group differences in the NTCB subtests were found between the AUT and AUT + ADHD groups.

**TABLE 2:** Mean (standard deviation) scores on subtests and composite indices of the NIH Toolbox Cognitive Battery by diagnostic group.

		NoAUTADHD	AUT	ADHD	AUT + ADHD		
Measure		(*N* = 737)	(*N* = 81)	(*N* = 162)	(*N* = 55)	*p*‐value^a^	Group differences
Fluid	DCCS	95.6 (18.4)	86.2 (18.9)	92.1 (18.6)	82.9 (18.6)	<0.001	AUT, AUT + ADHD < NoAUTADHD, AUT + ADHD < ADHD
	Flanker	83.1 (12.6)	82.3 (15.1)	83.3 (15.7)	79.2 (14.5)	0.37	
	LSWM	99.2 (14.9)	93.8 (18.5)	95.4 (16.2)	94.3 (15.5)	0.002	AUT < NoAUTADHD
	PCPS	96.2 (24.0)	86.2 (22.2)	96.5 (25.0)	85.8 (27.3)	<0.001	AUT, AUT + ADHD < NoAUTADHD, AUT < ADHD
	PSM	100.5 (16.9)	100.0 (20.7)	98.5 (16.4)	94.5 (16.9)	0.17	
Crystallized	ORT	104.0 (18.6)	99.5 (17.6)	101.4 (18.5)	97.7 (18.8)	0.06	
	PV	99.9 (15.1)	96.1 (16.9)	100.1 (16.7)	98.6 (14.7)	0.37	
Fluid Composite Index		91.6 (18.6)	83.2 (20.0)	88.7 (20.3)	79.5 (18.8)	<0.001	AUT, AUT + ADHD < NoAUTADHD, AUT + ADHD < ADHD
Crystallized Composite Index		102.3 (17.3)	97.6 (18.0)	100.8 (18.8)	97.9 (16.8)	0.17	
						0.13 (interaction)^b^	

Abbreviations: ADHD, attention‐deficit/hyperactivity disorder; AUT, autism spectrum disorder; AUT + ADHD, co‐occurring autism and ADHD; DCCS, Dimensional Card Change Sort; LSWM, List‐Sorting Working Memory; NoAUTADHD, no autism or ADHD; ORT, Oral Reading Test; PCPS, Pattern Comparison Processing Speed; PSM, Picture Sequence Memory; PV, Picture Vocabulary; TYP, typical development.

^a^ One‐way analysis of variance (ANOVA) with Holm‐Bonferroni correction for multiple testing.

^b^ Unmatched *p‐*value (interaction) = (Diagnosis: ADHD vs. AUT vs. AUT + ADHD vs. TYP) × 2 (Cognition: Crystallized vs. Fluid) factorial ANOVA.

### Associations between diagnostic groups and NTCB Fluid and Crystalized Composite scores

The associations between diagnostic groups and age‐adjusted Composite indices are displayed in Table 3. The Fluid Composite Index scores for the AUT + ADHD (*β* = −10.1; 95% confidence interval [CI] −14.8, −5.3, Cohen's *d* = −0.54) and AUT (*β* = −5.2; 95% CI −9.8, −0.6, Cohen's *d* = −0.28) groups were lower than the NoAUTADHD group. There was no evidence of an interaction between IQ score and diagnostic group for the Fluid Cognition Index scores (*p* = 0.94). To confirm the robustness of the findings, we repeated the analysis without the IQ × Group interaction term, and the results remained unchanged, indicating that the observed group differences in Fluid Composite Index scores were not moderated by IQ. When stratifying by sex, significantly lower Fluid Cognition Index scores were observed for the AUT + ADHD group compared with the NoAUTADHD group for both females (*β* = −12.7; 95% CI −21.7, −3.8) and males (*β* = −9.06; 95% CI −14.80, −3.35; Table S1). We found no interaction between AUT and ADHD diagnostic status (*p* = 0.26) (not displayed).

**TABLE 3:** Associations between fluid and crystallized indices and diagnostic groups.

	With IQ × group interaction	
	*β* (95% CI)	*p*‐value
Age‐adjusted Fluid Composite Index		
NoAUTADHD	Reference	
AUT	−5.2 (−9.8, −0.6)	0.03
ADHD	−1.4 (−4.3, 1.5)	0.36
AUT + ADHD	−10.1 (−14.8, −5.3)	<0.001
IQ	0.6 (0.5, 0.7)	<0.001
IQ × group^a^		0.94 (interaction)
Age‐adjusted Crystallized Composite Index		
NoAUTADHD	Reference	
AUT	−1.9 (−5.5, 1.74)	0.31
ADHD	−0.9 (−3.2, 1.4)	0.43
AUT + ADHD	−3.7 (−7.5, 0.04)	0.05
IQ	0.7 (0.6, 0.8)	<0.001
IQ × group^a^		0.26 (interaction)

Abbreviations: ADHD, attention‐deficit/hyperactivity disorder; AUT, autism spectrum disorder; AUT + ADHD, co‐occurring autism and ADHD; CI, confidence interval; IQ, intelligence quotient; NoAUTADHD, no autism or ADHD.

^a^ Likelihood ratio test for interaction.

### NTCB cognitive profiles

The fit statistics for LPA models generated from our IQ‐matched samples with two to five classes are shown in Table S2. The AIC and BIC values decreased steadily as the number of classes increased; however, we chose the four‐class model as the best model as it was the most conservative model with a sufficient sample in each class. While the five‐class model demonstrated slightly lower AIC (14,141.09 vs. 14,192.88) and BIC (14,301.77 vs. 14,325.61) values and marginally higher entropy (0.79 vs. 0.82) relative to the five‐class model, the improvement was minimal. Therefore, the four‐class model was selected to avoid adding complexity without significant benefit (Nylund et al., 2007). One‐way ANOVA with Holm‐Bonferroni correction for multiple testing showed significant differences between LPA classes for all NTCB subtests and Composite indices (*p* < 0.001).

**FIGURE 1:**
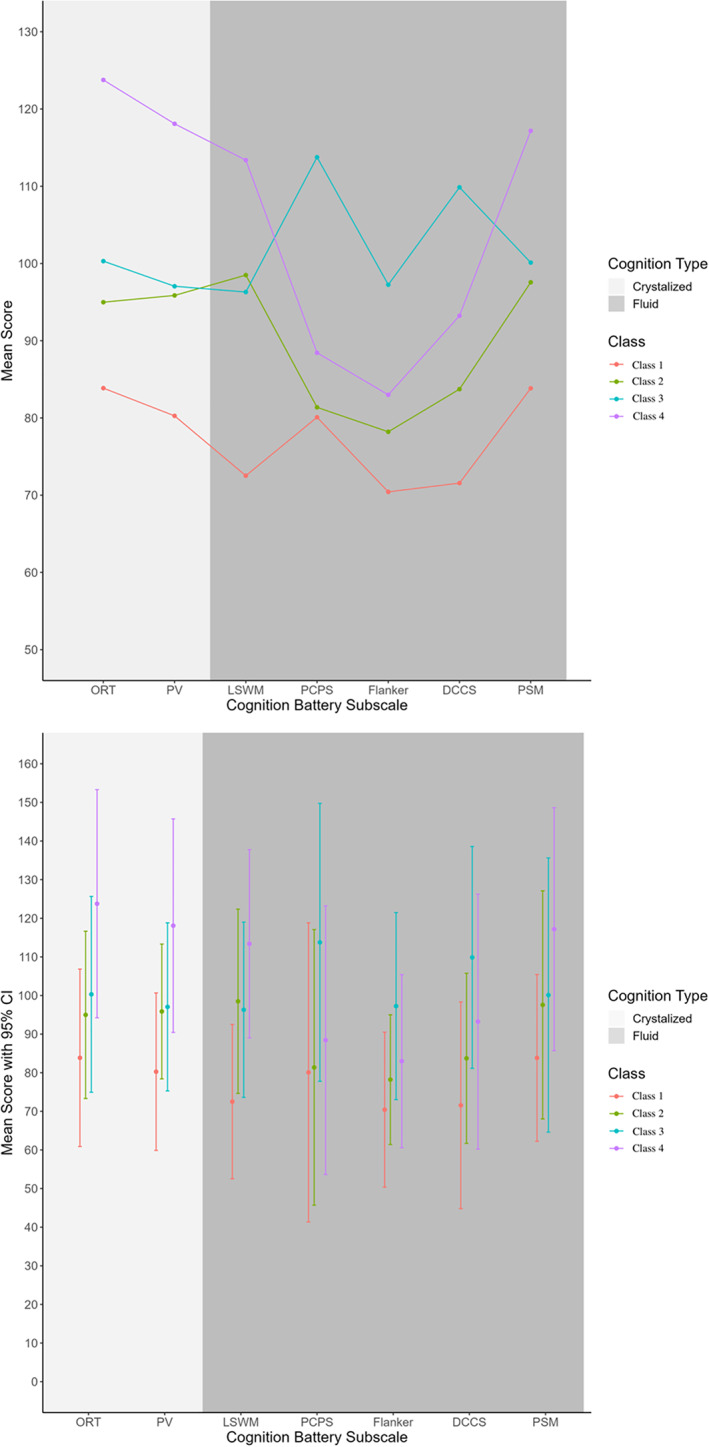
Profiles for four executive control groups by subtests of the National Institutes of Health Toolbox Cognition Battery.

Table S3 shows the descriptive characteristics of each LPA class and Table S4 shows sociodemographic characteristics of this IQ‐matched sample. Table 4 shows the differences in mean (SD) subtest scores, and Figure 1 illustrates the different cognitive profiles of each LPA class, including the mean subtest scores by each LPA Class, which is also shown in Table S5. Class 1 comprised 18.1% of the total sample and was characterized by the lowest scores overall (IQ, NTCB Composites and subtests). Class 1 included 21% of the AUT group, 19% of the ADHD group, and 15% of the NoAUTADHD group. Class 2 was the largest group (37% of the total sample) and had average mean IQ and Crystallized Composite scores and a low average Fluid Composite score. Class 2 also showed varying performance across subtests, with scores ranging from 78.2 (Flanker) to 98.5 (LSWM). The highest percentage of NoAUTADHD persons were assigned to Class 2 (41%), which also included 36% of the AUT group and 35% of the ADHD group. Class 3 included 30.9% of the sample and had average IQ, Fluid, and Crystalized scores. Class 3 demonstrated the highest scores in set shifting (DCCS: *M* = 109.9), inhibition (Flanker: *M* = 97.3), and processing speed (PCPS: *M* = 113.8). The highest percentage of persons with ADHD were assigned to Class 3 (36%), which also included 32% of the NoAUTADHD group and 25% of the AUT group. Class 4 group accounted for 14% of the sample and had the highest mean IQ score and the greatest disparity between Crystallized and Fluid Composite Indices (124 vs. 98). Class 4 had the highest scores in receptive vocabulary (PV: *M* = 118.1), oral reading (ORT: *M* = 123.8), episodic memory (PSM: *M* = 117.2) and working memory (LSWM: *M* = 113.4), despite low average performance in set shifting and inhibition. Class 4 included 19% of the AUT group, 12% of the NoAUTADHD group, and 11% of the ADHD group.

**TABLE 4:** Mean (standard deviation) scores on subtests and composite indices of the NIH Toolbox Cognitive Battery by four LPA classes.

	Class 1	Class 2	Class 3	Class 4	*p*‐value^a^
IQ‐matched sample					
*N* (%)	44 (18.1)	90 (37.0)	75 (30.9)	34 (13.4)	
DCCS	71.6 (13.7)	83.7 (11.2)	109.9 (14.7)	93.2 (16.9)	<0.001
Flanker	70.4 (10.2)	78.2 (8.6)	97.3 (12.4)	83.0 (11.4)	<0.001
LSWM	72.5 (10.2)	98.5 (12.2)	96.3 (11.6)	113.4 (12.4)	<0.001
PCPS	80.1 (19.8)	81.4 (18.2)	113.8 (18.4)	88.4 (17.7)	<0.001
PSM	83.8 (11.0)	97.6 (15.1)	100.1 (18.1)	117.2 (16.0)	<0.001
ORT	83.9 (11.7)	95.0 (11.1)	100.3 (12.9)	123.8 (15.1)	<0.001
PV	80.3 (10.4)	95.9 (8.9)	97.1 (11.1)	118.1 (14.1)	<0.001
Fluid Composite Index	60.9 (11.4)	80.3 (9.5)	105.2 (14.2)	98.2 (12.9)	<0.001
Crystalized Composite Index	79.2 (9.6)	94.7 (9.2)	98.5 (11.6)	124.5 (14.2)	<0.001

Abbreviations: ANOVA, analysis of variance; DCCS, Dimensional Card Change Sort; LPA, latent profile analysis; LSWM, List‐Sorting Working Memory; NIH, National Institutes of Health; ORT, Oral Reading Test; PCPS, Pattern Comparison Processing Speed; PSM, Picture Sequence Memory; PV, Picture Vocabulary.

^a^One‐way ANOVA with Holm‐Bonferroni correction for multiple testing.

### Sensitivity analyses

Sociodemographic characteristics of the sample without the NICU cohort can be found in Table S4. Sensitivity analyses comparing the ANOVA with and without the NICU cohort revealed that the Fluid (*p* < 0.001) and Crystallized (*p* = 0.70) Composite Index scores were consistent with the results for the full sample. However, in the subsample excluding the NICU cohort, Flanker scores for the Fluid Composite Index were significantly different between diagnostic groups (*p* = 0.02), with AUT + ADHD children having lower scores than the ADHD children (Table S5). The GLM analysis indicated that AUT children performed significantly lower on the Fluid Composite Index (*β* = −7.0; 95% CI: −11.5, −2.4), consistent with the findings when the NICU cohort was included (Table S6). However, when the NICU cohort was excluded, AUT children also showed significantly lower scores on the Crystallized Composite Index (*β* = −4.5; 95% CI: −8.3, −0.7). The results for the sex‐stratified analyses excluding the NICU cohort were consistent with the sex‐stratified analysis for the entire sample (Table S7).

## DISCUSSION

The primary purpose of this study was to replicate and extend prior findings from Solomon et al. (2021) that suggested poorer performance in fluid, but not crystallized, cognition in autistic youth relative to youth without autism using data from five cohorts in the nationwide multi‐site ECHO Program. With this larger sample, we also sought to examine the role of co‐occurring ADHD. This study confirmed previous findings (Solomon et al., 2021) that autistic youth perform worse in fluid cognition tasks on the NTCB relative to individuals without autism, but comparably in crystallized/verbal tasks. We further showed that this pattern extends to autistic youth with comorbid ADHD and that the presence of an ADHD diagnosis may not significantly worsen cognitive performance on the NTCB among autistic youth compared with having autism alone. Similar to Solomon et al. (2021), the LPA indicated that a significant proportion of autistic youth belonged to a class with a lower Fluid Composite Index score than Crystallized Composite index score. However, there was a substantial amount of overlap between diagnostic groups within each LPA class, suggesting that cognitive profiles on the NTCB may span across populations of those with and without autism or ADHD.

Group differences in the NTCB subtests were largely consistent with prior literature on cognitive functioning among autistic individuals. For example, relative to their peers without autism or ADHD, the AUT and AUT + ADHD groups scored lower in cognitive flexibility (DCCS), a pattern that aligns with previous findings (Ozonoff et al., 2004; Yerys et al., 2009). AUT children also performed lower in working memory (LSWM), and AUT + ADHD children performed lower in processing speed (PCPS), relative to their peers without autism or ADHD, which is in line with previous studies that have documented distinct cognitive profiles in individuals with autism using the Wechsler series of intelligence tests (Braconnier & Siper, 2021; Takayanagi et al., 2021; Wilson, 2024). The absence of group differences in the Flanker subtest was somewhat surprising, given the well‐known differences in response inhibition among autistic individuals (Geurts et al., 2014). Interestingly, group differences in the Flanker subtest were found when the NICU cohort was excluded, with AUT + ADHD children having lower scores than those with ADHD only. This may indicate that the inclusion of the NICU cohort confounded our findings; however, this is unlikely given that when the NICU cohort was excluded from the analyses, the NoAUTADHD group (IQ mean = 101.1) still performed below average on the Flanker subtest (score = 86.0). The NTCB Flanker subtest has previously been reported to have weak construct validity when examined in conjunction with other neuropsychological tests of attention and cognition in a sample of healthy adults (Ott et al., 2022); thus, further research is necessary to evaluate the validity of this subtest. Nonetheless, our finding of group differences in the Fluid Composite Index score and several Fluid subtest scores on the NTCB corroborates prior evidence of cognitive difficulties in autistic individuals, which has been proposed to underlie the theory of mind (Ozonoff et al., 1991; Pellicano, 2010) and restricted and repetitive behaviors (Iversen & Lewis, 2021) associated with the disorder.

The lack of group differences in the NTCB Crystallized Composite Index and subtest scores is consistent with prior findings among autistic individuals. Autistic children have been shown to exhibit average to above average scores in verbal comprehension (Mayes & Calhoun, 2008) and word reading (Gabig, 2010; Nation et al., 2006; Newman et al., 2007). Thus, continued research is warranted to examine relative cognitive strengths in autistic persons in contrast to the deficit‐based approach that has dominated research in the field (Bottema‐Beutel et al., 2021).

One of our objectives was to investigate the impact of ADHD on cognitive abilities in autistic youth. Our findings showed no significant differences between the AUT and AUT + ADHD groups on NTCB subtests. When controlling for age and IQ, AUT + ADHD children had significantly lower Fluid Composite Index scores than NoAUTADHD children, whereas AUT children had only slightly lower scores than NoAUTADHD children; however, no interaction was observed between AUT and ADHD diagnostic status. Moreover, when stratifying the analyses by sex, both males and females from the AUT + ADHD group had significantly lower Fluid Composite Index scores than the NoAUTADHD group, which is consistent with a recent study that did not find evidence of ADHD‐related sex differences on the NTCB (Assari, 2021). Altogether, our findings indicate a limited potential impact of ADHD on cognitive abilities in autistic youth. This is in line with the non‐significant correlation between NTCB scores and parent‐reported ADHD symptoms found by Solomon et al. (2021) but is in contrast to other studies indicating that ADHD symptoms exacerbate cognitive functioning in autistic individuals (Rosello et al., 2022; Yerys et al., 2009). While the NTCB was designed to provide a standard measure of cognitive abilities with minimized floor and ceiling effects, the NTCB may not be sensitive to cognitive impairments associated with ADHD. For example, traditional neuropsychological tasks that target ADHD, such as a continuous performance task, are intentionally long in duration (≈15 min) to tap into sustained attention and vigilance, whereas NTCB tasks are brief (3–7 min). This may also explain why the ADHD only group performed similarly to the group without autism or ADHD on the Fluid Composite Index.

The results from the LPA analyses were somewhat similar to the findings from Solomon et al. (2021). First, we found that a large percentage (19%) of AUT participants were classified in Class 4, which had Crystallized Composite scores that were nearly two SDs above the mean with a 25+ point discrepancy between Crystallized and Fluid Composite scores. Second, as before (Solomon et al., 2021), we found that the class with the lowest scores on both indices (Class 1) had the fewest NoAUTADHD participants and that most NoAUTADHD participants belonged to the group with low average Fluid and average Crystallized Composite scores (Class 2). Third, and somewhat surprisingly, a large proportion of the participants with ADHD only were classified into the class with average Fluid and Crystallized Composite scores, average IQs, and a minimal Fluid Cognition to Crystalized Cognition discrepancy (Class 3). Importantly, both clinical and NoAUTADHD participants were classified into each of the LPA groups, suggesting considerable transdiagnostic overlaps in the cognitive profiles of youth. This suggests that cognitive patterns may be found more broadly than just in autism and that diagnostic patterns become less pronounced when working with a cross‐diagnostic sample. This also highlights the fact that not all individuals with neurodevelopmental conditions experience the same cognitive challenges and that there are some individuals with average to above average cognitive abilities, highlighting the importance of transdiagnostic analytical approaches in addition to group‐level analyses.

Although our study has several strengths, including a large, diverse nationwide sample, we acknowledge several limitations. First, as these data were a pooled analysis of secondary data obtained from ongoing cohort studies, autism and ADHD status were determined based on parent report of a clinician diagnosis. Given that the co‐occurrence of autism and ADHD has only been formally recognized in the most recent revision of the Diagnostic and Statistical Manual of Mental Disorders (American Psychological Association, 2013), it is possible that some autistic individuals experience significant ADHD symptoms but have not been assessed and diagnosed with ADHD. Thus, future work may benefit from outcome ascertainment through gold‐standard diagnostic assessments. Second, one of the cohorts included in this study consists mostly of youth who were born extremely premature, which may limit the generalizability of the study's findings. Sensitivity analyses indicated, however, that the results with and without the extremely premature cohort were largely similar (see Tables S4–S8). Third, our comparison group may include children who are not neurotypical as they may have other neurodevelopmental conditions (e.g., Tourette syndrome/tics, language difficulties, dyspraxia, dyslexia) that were not assessed and future research investigation the effects of these conditions are warranted. We also limited our study to those with IQs > 70 and therefore our results may not generalize to individuals with intellectual disability. Fourth, although our goal was to replicate the original findings in a larger community sample, we acknowledge the reduced experimental control (i.e., varied IQ battery across sites) that more representative designs often require in the name of external over internal validity (e.g., Handley et al., 2018). Finally, while we believe it was important to compare individuals without autism or ADHD who had similar IQ scores to those with these disorders, the IQ‐matched sample used in the LPA was relatively small, which may limit the generalizability of the LPA findings.

In conclusion, the findings from this study indicate that autistic persons with or without ADHD most commonly perform worse on fluid cognition tasks than on crystallized/verbal tasks on the NTCB. Furthermore, we found that the cognitive profiles identified by the LPA in this sample of three diagnostic groups were similar to those found in Solomon et al. (2021), with only two diagnostic groups. However, relative to Solomon et al. (2021), the general pattern was toward more similarities in group assignment than differences, highlighting that cognitive profiles may be transdiagnostic. Thus, interventions that target cognitive functions should be based on individual cognitive performance rather than diagnostic status. For example, not all individuals with autism or ADHD will benefit from working memory training. Lastly, given that a subset of autistic persons demonstrated cognitive functioning that fell within the average range and superior verbal abilities, a strength‐based approach to research in cognition among those with neurodevelopmental disorders is encouraged.

## AUTHOR CONTRIBUTIONS

**Catrina A. Calub:** Conceptualization; methodology; writing—original draft; writing—review and editing. **Aisha S. Dickerson:** Conceptualization; methodology; writing—review and editing. **Haozuo Zhao:** Conceptualization; data curation; formal analysis; methodology; visualization; writing—review and editing. **Robert M. Joseph:** Conceptualization; investigation; methodology; writing—review and editing. **Michael O'Shea:** Conceptualization; funding acquisition; investigation; methodology; resources; writing—review and editing. **Shaikh I. Ahmad:** Investigation; writing—review and editing. **Lisa A. Croen:** Investigation; writing—review and editing. **Sean C. L. Deoni:** Investigation; writing—review and editing. **Viren A. D’Sa:** Investigation; writing—review and editing. **Akhgar Ghassabian:** Investigation; writing—review and editing. **Daphne Koinis‐Mitchell:** Funding acquisition; resources; investigation; writing—review and editing. **Leonardo Trasande:** Funding acquisition; resources; investigation; writing—review and editing. **Heather Volk:** Conceptualization; writing—review and editing. **Anna J. Yeo:** Investigation; writing—review and editing. **Irva Hertz‐Picciotto:** Conceptualization; funding acquisition; resources; investigation; methodology; writing—review and editing. **Julie B. Schweitzer:** Conceptualization; funding acquisition; resources; investigation; methodology; writing—review and editing. **Marjorie Solomon:** Conceptualization; investigation; methodology; writing—original draft; supervision.

## CONFLICT OF INTEREST STATEMENT

The authors declare no conflicts of interest.

## ETHICAL CONSIDERATIONS

The study protocol was approved by the single ECHO institutional review board, WCG IRB. Written informed consent or parent's/guardian's permission was obtained along with child assent as appropriate, for the ECHO Cohort Data and Biospecimen Collection Protocol participation and for participation in specific study sites.

## Supporting information

Supporting Information S1

Supporting Information S2

## Data Availability

Select de‐identified data from the ECHO Program are available through NICHD's%20Data%20and%20Specimen%20Hub%20(DASH). Information on study data not available on DASH, such as some Indigenous datasets, can be found on the ECHO%20study%20DASH%20webpage.
