# Supplementary material for: Assessing cognition in autistic youth with and without attention‐deficit/hyperactivity disorder using the NIH Toolbox Cognition Battery: An Environmental influences on Child Health Outcomes‐Wide Cohort Study
Source: JCPP Adv. 2025 Jul 16;6(2):e70031. doi: 10.1002/jcv2.70031 (PMC13260678; doi:10.1002/jcv2.70031)
Supplement: Supplementary file 1 — Supporting Information S1 [file JCV2-6-e70031-s001.docx]

**SUPPORTING INFORMATION**

**Figure S1.** Participant Flowchart

Participants with NIH Toolbox Scores

N = 11,701 children

N = 58 cohorts

Missing Fluid Cognition Score = 6,491

Missing Crystalized Cognition Score = 6,353

n = 6,491 children

n = 15 cohorts

Participants with Cognition Indexes

n = 5,210 children

n = 43 cohorts

Not 12-18 Years of Age

n = 3,266 children

n= 20 cohorts

Participants 12-18 Years of Age

n = 1,944

n = 23 cohorts

Missing Total NIH Composite Score or Subtest Score

n = 25 children

Included in Analysis

n = 1,919

n = 23 cohorts

Missing ASD or ADHD Status

n = 271 children

n = 2 cohorts

Included in Analysis

n = 1,648

n = 21 cohorts

Missing IQ or IQ score <70

n = 613 children

n = 16 cohorts

**Included in final analysis**

**N = 1,035 children**

**N = 5 cohorts**

ADHD, attention-deficit/hyperactivity disorder; AUT, autism spectrum disorder; IQ, intelligence quotient; NIH, National Institutes of Health.

**Table S1**

*Sex-Stratified Analysis of Associations Between Age-Adjusted Fluid and Crystallized Indices and Diagnostic Groups*

|  | **Female  (n=480)** | | **Male  (n=555)** | | **Overall  (n=1035)** | |
| --- | --- | --- | --- | --- | --- | --- |
| **Group** | **β (95% CI)** | ***P*-value** | **β (95% CI)** | ***P*-value** | **β (95% CI)** | ***P*-value** |
| **Age-adjusted Fluid Composite Index** | | | | |  |  |
| NoAUTADHD | Ref |  | Ref |  | Ref |  |
| AUT | -3.3 (-13.7, 7.2) | 0.54 | -4.6 (-9.8, 0.6) | 0.08 | -5.2 (-9.8, -0.6) | 0.03 |
| ADHD | -3.5 (-8.2, 1.2) | 0.14 | -0.5 (-4.2, 3.2) | 0.79 | -1.4 (-4.3, 1.5) | 0.36 |
| AUT+ADHD | -12.7 (-21.7, -3.8) | 0.01 | -9.1 (-14.8, -3.4) | <0.001 | -10.1 (-14.8, -5.3) | <0.001 |
| IQ | 0.6 (0.5, 0.7) | <0.001 | 0.5 (0.4, 0.7) | <0.001 | 0.6 (0.5, 0.7) | <0.001 |
| **Age-adjusted Crystalized Composite Index** | | | | |  |  |
| NoAUTADHD | Ref |  | Ref |  | Ref |  |
| ADHD | -1.8 (-10.2, 6.7) | 0.68 | -1.8 (-5.8, 2.3) | 0.39 | -1.9 (-5.5, 1.7) | 0.31 |
| AUT | -1.5 (-5.3, 2.3) | 0.43 | -1.2 (-4.1, 1.7) | 0.42 | -0.9 (-3.2, 1.4) | 0.43 |
| AUT+ADHD | -6.8 (-14.1, 0.4) | 0.06 | -2.8 (-7.2, 1.7) | 0.23 | -3.7 (-7.5, 0.04) | 0.05 |
| IQ | 0.7 (0.6, 0.8) | <0.001 | 0.7 (0.6, 0.8) | <0.001 | 0.7 (0.6, 0.8) | <0.001 |

^a^Likelihood ratio test for interaction.

ADHD, attention-deficit/hyperactivity disorder; AUT, autism spectrum disorder; CI, confidence interval; NoAUTADHD, no autism or ADHD.

**Table S2**

*Model Fit Statistics and Class Probabilities for LPA Models With Two to Five Classes*

| Number of classes | AIC | BIC | Entropy | PBLRT p-value |
| --- | --- | --- | --- | --- |
| Unmatched sample | | | | |
| Two | 61,024.67 | 61,133.40 | 0.69 | <0.001 |
| Three | 60,751.91 | 60,900.17 | 0.71 | <0.001 |
| Four | 60,516.17 | 60,703.97 | 0.72 | <0.001 |
| Five | 60,374.19 | 60,601.53 | 0.71 | <0.001 |
| Full IQ-matched sample | | | | |
| Two | 14,355.33 | 14,432.18 | 0.73 | <0.001 |
| Three | 14,255.47 | 14,360.26 | 0.79 | <0.001 |
| Four | 14,192.88 | 14,325.61 | 0.79 | <0.001 |
| Five | 14,141.09 | 14,301.77 | 0.82 | <0.001 |
| Subsample without NICU cohort | | | | |
| Two | 11,331.07 | 11,402.73 | 0.74 | <0.001 |
| Three | 11,265.45 | 11,363.17 | 0.86 | <0.001 |
| Four | 11,223.95 | 11,347.73 | 0.79 | <0.001 |
| Five | 11,178.74 | 11,328.59 | 0.85 | <0.001 |

*Note*: Lower numbers for AIC, BIC, and aBIC indicate more optimal model fit. Small *p*-values of the LMRT and PBLRT tests indicate that the model with a greater number of classes fits the data better than the previous model. Entropy closer to 1 indicates that the children are well-categorized into classes. The final model is bolded for emphasis.
AIC, Akaike Information Criterion; BIC, Bayesian Information Criterion; LPA, latent profile analysis; PBLRT, Parametric Bootstrapped Likelihood Ratio Test.

**Table S3**

*Descriptive Statistics and Scores on Composite Indices of the NIH Toolbox Cognitive Battery by Diagnostic Status for Four Executive Control (EC) Groups*

|  | **Class 1** | **Class 2** | **Class 3** | **Class 4** | ***P-*value** |
| --- | --- | --- | --- | --- | --- |
| **Unmatched Sample** | | | | | |
| N (%) | **357 (34.5)** | **288 (27.8)** | **282 (27.2)** | **108 (10.4)** |  |
| Age (years), mean (SD) | 15.4 (2.0) | 15.0 (2.2) | 14.9 (1.7) | 15.0 (2.0) | 0.001 |
| Sex, n (%)^b^ |  |  |  |  |  |
| Male | 178 (49.9%) | 158 (54.9%) | 162 (57.4%) | 57 (52.8%) | 0.27 |
| Female | 179 (50.1%) | 130 (45.1%) | 120 (42.6%) | 51 (47.2%) |  |
| IQ, mean (SD)^a^ | 93.2 (12.1) | 99.6 (11.8) | 109.5 (11.8) | 101.3 (13.9) | <0.001 |
| Group, n (%)^b^ |  |  |  |  | 0.003 |
| NoAUTADHD | 226 (63.3%) | 217 (75.3%) | 210 (74.5%) | 84 (77.8%) |  |
| AUT | 36 (10.1%) | 22 (7.6%) | <20 | <5 |  |
| ADHD | 65 (18.2%) | 38 (13.2%) | 40 (14.2%) | 19 (17.6%) |  |
| **IQ-matched sample** | | | | | |
| N (%) | **44 (18.1)** | **90 (37.0)** | **75 (30.9)** | **34 (14.0)** |  |
| Age (years), mean (SD) | 15.2 (2.1) | 15.2 (2.1) | 14.8 (2.3) | 14.5 (1.8) | 0.34 |
| Sex, n (%)^b^ |  |  |  |  |  |
| Male | 24 (54.5%) | 61 (67.8%) | 53 (70.7%) | 26 (76.5%) | 0.18 |
| Female | 20 (45.5%) | 29 (32.2%) | 22 (29.3%) | 8 (23.5%) |  |
| IQ, mean (SD)^a^ | 82.2 (8.7) | 94.4 (11.4) | 97.0 (11.1) | 111.5 (10.3) | <0.001 |
| Group, n (%)^b^ |  |  |  |  | 0.57 |
| NoAUTADHD | 12 (14.8%) | 33 (40.7%) | 26 (32.1%) | 10 (12.3%) |  |
| AUT | 17 (21.0%) | 29 (35.8%) | 20 (24.7%) | 15 (18.5%) |  |
| ADHD | 15 (18.5%) | 28 (34.6%) | 29 (35.8%) | 9 (11.1%) |  |
| **IQ-matched sample without NICU cohort** | | | | | |
| N (%) | **23 (12.0)** | **81 (42.2)** | **60 (31.3)** | **28 (14.5)** |  |
| Age (years), mean (SD) | 14.7 (2.64) | 15.0 (2.29) | 14.8 (2.44) | 14.4 (1.97) | 0.63 |
| Sex, n (%)^b^ |  |  |  |  |  |
| Male | 15 (65.2%) | 56 (69.1%) | 42 (70.0%) | 22 (78.6%) | 0.74 |
| Female | 8 (34.8%) | 25 (30.9%) | 18 (30.0%) | 6 (21.4%) |  |
| IQ, mean (SD)^a^ | 85.7 (9.37) | 95.3 (10.8) | 97.7 (11.0) | 113 (9.56) | <0.001 |
| Group, n (%)^b^ |  |  |  |  | 0.53 |
| NoAUTADHD | 7 (10.9.4%) | 28 (43.8%) | 21 (32.8%) | 8 (12.5%) |  |
| AUT | 9 (14.1%) | 29 (45.3%) | 14 (21.9%) | 12 (18.8%) |  |
| ADHD | 7 (10.9%) | 24 (37.5%) | 25 (39.1%) | 8 (12.5%) |  |

*Note*. In both the unmatched and IQ-matched samples, the cognitive profiles share some similarities. Both include a low cognitive profile class (EC1 in both samples), an average class (EC2 in the unmatched sample and EC3 in the IQ-matched sample), and a group with very high crystallized but average fluid scores (EC3 in the unmatched sample and EC4 in the IQ-matched sample). Notably, the unmatched sample also features a group with average IQ but very high fluid and crystallized scores (EC4), whereas the IQ-matched sample includes a class (EC2) with average IQ and crystallized scores but lower fluid scores. The unmatched sample is larger and includes individuals with higher average IQ, fluid, and crystallized scores across all classes. For instance, the IQ for EC1 in the unmatched sample is 93.2 (SD = 11.2), compared to 82.2 (SD = 8.7) in the IQ-matched sample. In the unmatched sample, individuals with autism and ADHD are predominantly concentrated in EC1, the class with the lowest cognitive scores (43.4% autism, 40.1% ADHD). In contrast, in the IQ-matched sample, individuals with autism (35.8%), ADHD (34.6%), as well as those without autism or ADHD (40.7%) are concentrated in EC2, which is characterized by average IQ and crystallized scores but lower fluid scores. In the unmatched sample, TD individuals are more evenly distributed across EC1 (30.0%), EC2 (28.5%), and EC3 (26.8%). This redistribution reflects the impact of removing TD individuals (excluded n = 656) who do not match the IQ of autistic youth.

Four classes differ significantly following post-hoc multiple comparison correction (Tukey's HSD).
^a^*P-*values based on one-way analysis of variance (ANOVA) with Holm-Bonferroni correction for multiple testing.
^b^*P-*value based on Chi-squared tests for statistical significance.
ADHD, attention-deficit/hyperactivity disorder; IQ, intelligence quotient; NICU, neonatal intensive care unit; SD, standard deviation.

**Table S4**

*Sociodemographic Characteristics of the Participating Children of IQ-matched Sample and Samples Excluding the NICU Cohort*

|  | **NoAUTADHD** | **AUT** | **ADHD** | **AUT+ADHD** | ***P-*value** |
| --- | --- | --- | --- | --- | --- |
| **IQ-matched sample** | **(n = 81)** | **(n = 81)** | **(n = 81)** |  |  |
| Sex, n (%) |  |  |  |  |  |
| Male | 49 (60.5%) | 61 (75.3%) | 54 (66.7%) |  | 0.129 |
| Female | 32 (39.5%) | 20 (24.7%) | 27 (33.3%) |  |  |
| Race |  |  |  |  |  |
| White | 43 (53.1%) | 60 (74.1%) | 53 (65.4%) |  | 0.001 |
| Black | 29 (35.8%) | 7 (8.6%) | 21 (25.9%) |  |  |
| Other/Multiple Race | 9 (11.1%) | 13 (16.0%) | 7 (8.6%) |  |  |
| Missing | 0 (0%) | 1 (1.2%) | 0 (0%) |  |  |
| Ethnicity |  |  |  |  |  |
| Hispanic | 12 (14.8%) | <22 (<27.2%) | <5 (<6.2%) |  | 0.006 |
| Non-Hispanic | 69 (85.2%) | <81 (<100%) | <81 (<100%) |  |  |
| Age (years), mean (SD) | 14.7 (2.2) | 15.1 (2.1) | 15.0 (2.2) |  | 0.474 |
| **Unmatched sample without NICU cohort** | **(N=426)** | **(N=64)** | **(N=80)** | **(N=35)** |  |
| Sex, n (%) |  |  |  |  |  |
| Male | 219 (51.4%) | 51 (79.7%) | 58 (72.5%) | 27 (77.1%) | <0.001 |
| Female | 207 (48.6%) | 13 (20.3%) | 22 (27.5%) | 8 (22.9%) |  |
| Race |  |  |  |  |  |
| White | 199 (46.7%) | 47 (73.4%) | 47 (58.8%) | 25 (71.4%) | <0.001 |
| Black | 179 (42.0%) | 5 (7.8%) | 25 (31.3%) | 5 (14.3%) |  |
| Other/Multiple Race | 47 (11.0%) | 12 (18.8%) | 8 (10.0%) | 5 (14.3%) |  |
| Missing | 1 (0.2%) | 0 (0%) | 0 (0%) | 0 (0%) |  |
| Ethnicity |  |  |  |  |  |
| Hispanic | 34 (8.0%) | 16 (25.0%) | <5 (<6.3%) | <9 (<25.7%) | <0.001 |
| Non-Hispanic | 392 (92.0%) | 48 (75.0%) | <80 (<100%) | <35 (<100%) |  |
| Age (years), mean (SD) | 14.7 (2.5) | 15.1 (2.3) | 14.8 (2.4) | 15.3 (2.3) | 0.511 |
| IQ score, mean (SD) | 101.1 (12.6) | 97.6 (13.0) | 98.4 (13.5) | 94.9 (13.8) | 0.008 |
| **IQ-matched sample without NICU cohort** | **(N=64)** | **(N=64)** | **(N=64)** |  |  |
| Sex, n (%) |  |  |  |  |  |
| Male | 39 (60.9%) | 51 (79.7%) | 45 (70.3%) |  | 0.068 |
| Female | 25 (39.1%) | 13 (20.3%) | 19 (29.7%) |  |  |
| Race |  |  |  |  |  |
| White | 34 (53.1%) | 47 (73.4%) | 40 (62.5%) |  | 0.005 |
| Black | 22 (34.4%) | 5 (7.8%) | 18 (28.1%) |  |  |
| Other/Multiple Race | 8 (12.5%) | 12 (18.8%) | 6 (9.4%) |  |  |
| Missing | 0 (0%) | 0 (0%) | 0 (0%) |  |  |
| Ethnicity |  |  |  |  |  |
| Hispanic | 11 (17.2%) | <19 (<29.7%) | <5 (<7.8%) |  | 0.006 |
| Non-Hispanic | 53 (82.8%) | <64 (<100%) | <64 (<100%) |  |  |
| Age (years), mean (SD) | 4.5 (2.3) | 15.1 (2.3) | 14.9 (2.4) |  | 0.323 |

*P-*values for differences between diagnostic groups are based on Chi-squared tests or Fisher’s exact tests of significance for sex, race, and ethnicity, and one-way analysis of variance (ANOVA) for age and IQ scores.
ADHD, attention-deficit/hyperactivity disorder; AUT, autism spectrum disorder; IQ, intelligence quotient; NICU, neonatal intensive care unit; SD, standard deviation.

**Table S5**

*Mean (Standard Deviation) Scores on Subtests and Composite Indices of the NIH Toolbox Cognitive Battery by Diagnostic Group of IQ-matched Sample and Samples Excluding the NICU Cohort*

| **Measure** | | | **NoAUTADHD** | **AUT** | **ADHD** | **AUT+ADHD** | ***P*-value^a^** |
| --- | --- | --- | --- | --- | --- | --- | --- |
| **IQ-matched sample** | | | **(N=81)** | **(N=81)** | **(N=81)** |  |  |
| Fluid DCCS | | | 92.4 (20.2) | 86.2 (18.9) | 94.2 (18.9) |  | 0.18 |
| Flanker | | | 83.2 (12.1) | 82.3 (15.1) | 84.6 (16.1) |  | 1.00 |
| LSWM | | | 97.6 (15.1) | 93.8 (18.5) | 94.3 (16.3) |  | 1.00 |
| PCPS | | | 93.0 (22.9) | 86.2 (22.2) | 97.1 (24.5) |  | 0.10 |
| PSM | | | 98.1 (17.6) | 100.0 (20.7) | 97.7 (16.0) |  | 1.00 |
| Crystallized ORT | | | 99.0 (16.1) | 99.5 (17.6) | 97.3 (17.1) |  | 1.00 |
| PV | | | 96.9 (13.1) | 96.1 (16.9) | 96.6 (15.1) |  | 1.00 |
| Fluid Composite Index | | | 88.6 (18.7) | 83.2 (20.0) | 89.4 (21.8) |  | 0.79 |
| Crystallized Composite Index | | | 97.6 (15.0) | 97.6 (18.0) | 96.5 (17.5) |  | 1.00 |
| **Unmatched sample without NICU cohort** | | | **(N=426)** | **(N=64)** | **(N=80)** | **(N=35)** |  |
| Fluid | | DCCS | 97.3 (18.5) | 88.3 (18.7) | 97.0 (18.9) | 78.9 (17.2) | <0.001 |
|  | | Flanker | 86.0 (12.9) | 84.7 (15.1) | 88.9 (16.7) | 78.5 (16.2) | 0.02 |
|  | | LSWM | 99.6 (15.0) | 95.1 (18.8) | 95.5 (17.3) | 92.6 (14.6) | 0.03 |
|  | | PCPS | 100.9 (23.0) | 86.9 (23.2) | 101.6 (21.8) | 83.9 (26.0) | <0.001 |
|  | | PSM | 100.6 (17.4) | 100.4 (20.6) | 99.5 (16.8) | 94.7 (17.6) | 0.70 |
| Crystallized | | ORT | 99.9 (17.4) | 98.2 (17.8) | 97.9 (18.5) | 92.3 (14.9) | 0.32 |
|  | | PV | 98.5 (15.1) | 96.9 (16.6) | 97.9 (15.9) | 96.0 (13.9) | 0.73 |
| Fluid Composite Index | | | 94.8 (18.9) | 85.6 (19.5) | 94.2 (20.4) | 77.1 (16.1) | <0.001 |
| Crystallized Composite Index | | | 99.1 (16.9) | 97.3 (18.6) | 97.6 (19.0) | 93.3 (14.6) | 0.70 |
|  | | |  |  |  |  |  |
| **IQ-matched sample without NICU cohort** | | | **(N=64)** | **(N=64)** | **(N=64)** |  |  |
| Fluid | DCCS | | 95.4 (20.3) | 88.3 (18.7) | 96.5 (18.6) |  | 0.28 |
|  | Flanker | | 84.8 (12.4) | 84.7 (15.1) | 87.6 (15.6) |  | 1.00 |
|  | LSWM | | 97.9 (14.1) | 95.1 (18.8) | 96.4 (16.2) |  | 1.00 |
|  | PCPS | | 96.3 (20.6) | 86.9 (23.2) | 101.0 (22.4) |  | 0.01 |
|  | PSM | | 100.0 (17.7) | 100.4 (20.6) | 98.8 (15.5) |  | 1.00 |
| Crystallized | ORT | | 99.3 (15.3) | 98.2 (17.8) | 98.3 (17.6) |  | 1.00 |
|  | PV | | 98.5 (12.8) | 96.9 (16.6) | 97.7 (15.3) |  | 1.00 |
| Fluid Composite Index | | | 91.5 (17.1) | 83.2 (20.0) | 93.5 (20.0) |  | 0.35 |
| Crystallized Composite Index | | | 98.7 (14.3) | 97.6 (18.0) | 97.7 (18.3) |  | 1.00 |
|  | | |  |  |  |  |  |

^a^One-way analysis of variance (ANOVA) with Holm-Bonferroni correction for multiple testing.

^b^Unmatched *p-*value (interaction) = (Diagnosis: ADHD vs AUT vs AUT+ADHD vs NoAUTADHD) x 2 (Cognition: Crystalized vs. Fluid) factorial ANOVA.

^c^Matched *p-*value (interaction) = (Diagnosis: ADHD vs AUT vs NoAUTADHD) x 2 (Cognition: Crystalized vs. Fluid) factorial ANOVA.

ADHD, attention-deficit/hyperactivity disorder; AUT, autism spectrum disorder; DCCS, Dimensional Card Change Sort; LSWM, List-Sorting Working Memory; NICU, neonatal intensive care unit; ORT, Oral Reading Test; PCPS, Pattern Comparison Processing Speed; PSM, Picture Sequence Memory; PV, Picture Vocabulary.

**Table S6**

*Associations Between Fluid and Crystallized Indices and Diagnostic Groups, Excluding the NICU Cohort*

|  | **With IQ * Group interaction** | |
| --- | --- | --- |
|  | **β (95% CI)** | ***P*-value** |
| **Age-adjusted Fluid Composite Index** | | |
| NoAUTADHD | Reference |  |
| AUT | -7.0 (-11.5, -2.4) | <0.001 |
| ADHD | 0.9 (-3.2, 5.0) | 0.68 |
| AUT+ADHD | -14.2 (-20.5, -8.0) | <0.001 |
| IQ | 0.7 (0.6, 0.8) | <0.001 |
| IQ * Group^a^ |  | 0.71 (interaction) |
| **Age-adjusted Crystalized Composite Index** | | |
| NoAUTADHD | Reference |  |
| AUT | -4.5 (-8.3, -0.7) | 0.02 |
| ADHD | 0.4 (-2.7, 3.4) | 0.8 |
| AUT+ADHD | -5.4 (-10.2, -0.7) | 0.03 |
| IQ | 0.8 (0.7, 0.9) | <0.001 |
| IQ * Group^a^ |  | 0.22 (interaction) |

^a^Likelihood ratio test for interaction.

ADHD, attention-deficit/hyperactivity disorder; AUT, autism spectrum disorder; CI, confidence interval; IQ, intelligence quotient; NICU, neonatal intensive care unit; NoAUTADHD, no autism or ADHD.

**Table S7**

*Sex-Stratified Analysis of Associations Between Age-Adjusted Fluid and Crystallized Indices and Diagnostic Groups, Excluding the NICU Cohort*

|  | **Female  (n = 250)** | | **Male  (n = 250)** | | **Overall  (n = 605)** | |
| --- | --- | --- | --- | --- | --- | --- |
| **Group** | **β (95% CI)** | ***P*-value** | **β (95% CI)** | ***P*-value** | **β (95% CI)** | ***P*-value** |
| **Age-adjusted Fluid Composite Index** | | | | |  |  |
| NoAUTADHD | Ref |  | Ref |  | Ref |  |
| AUT | -5.8 (-16.5, 5.1) | 0.30 | -6.9 (-12.2, -1.7) | 0.01 | -6.9 (-11.5, -2.4) | <0.001 |
| ADHD | -2.9 (-10.7, 4.9) | 0.46 | 1.9 (-3.1, 6.9) | 0.45 | 0.9 (-3.2, 5.0) | 0.68 |
| AUT+ADHD | -17.6 (-33.0, -2.1) | 0.03 | -14.0 (-21.0, -7.0) | <0.001 | -14.2 (-20.5, -8.0) | <0.001 |
| IQ | 0.7 (0.5, 0.9) | <0.001 | 0.7 (0.5, 0.9) | <0.001 | 0.7 (0.5, 0.8) | <0.001 |
| **Age-adjusted Crystalized Composite Index** | | | | |  |  |
| NoAUTADHD | Ref |  | Ref |  | Ref |  |
| ADHD | -7.5 (-16.1, 1.1) | 0.09 | -3.3 (-7.6, 0.9) | 0.13 | -4.5 (-8.3, -0.7) | 0.02 |
| AUT | 1.9 (-3.7, 7.6) | 0.49 | -0.4 (-4.1, 3.3) | 0.83 | 0.4 (-2.7, 3.4) | 0.80 |
| AUT+ADHD | -11.3 (-22.6, -0.03) | 0.05 | -4.3 (-9.8, 1.1) | 0.12 | -5.4 (-10.2, -0.7) | 0.03 |
| IQ | 0.8 (0.7, 0.9) | <0.001 | 0.8 (0.7, 0.9) | <0.001 | 0.8 (0.7, 0.9) | <0.001 |

^a^Likelihood ratio test for interaction.

ADHD, attention-deficit/hyperactivity disorder; AUT, autism spectrum disorder; CI, confidence interval; IQ, intelligence quotient; NICU, neonatal intensive care unit; NoAUTADHD, no autism or ADHD.

**Table S8**

*Mean (Standard Deviation) Scores on Subtests and Composite Indices of the NIH Toolbox Cognitive Battery by Four Executive Control (EC) Groups of Unmatched Sample with and without the NICU Cohort*

|  | **Class 1** | **Class 2** | **Class 3** | **Class 4** |  |
| --- | --- | --- | --- | --- | --- |
|  |  |  |  |  |  |
| **Unmatched Sample** |  |  |  |  |  |
| **N (%)** | **357 (34.4)** | **288 (27.8)** | **282 (27.2)** | **108 (10.4)** | ***P-*value** |
| DCCS | 78.1 (11.8) | 103.6 (12.4) | 91.4 (12.1) | 124.0 (12.5) | <0.001 |
| Flanker | 73.8 (9.4) | 90.7 (10.5) | 79.5 (8.9) | 101.0 (12.0) | <0.001 |
| LSWM | 88.3 (13.4) | 96.8 (12.1) | 106.0 (13.9) | 112.1 (12.6) | <0.001 |
| PCPS | 81.8 (20.0) | 109.2 (19.1) | 88.7 (20.7) | 116.6 (25.1) | <0.001 |
| PSM | 91.9 (13.7) | 98.7 (14.6) | 103.5 (16.8) | 119.5 (17.0) | <0.001 |
| ORT | 90.3 (12.3) | 96.8 (12.6) | 119.7 (15.2) | 117.0 (15.1) | <0.001 |
| PV | 88.9 (10.7) | 94.9 (10.7) | 113.3 (12.7) | 111.6 (11.5) | <0.001 |
| Fluid Composite Index | 72.3 (11.0) | 99.3 (9.4) | 89.7 (12.4) | 123.2 (12.3) | <0.001 |
| Crystalized Composite Index | 88.0 (10.6) | 95.2 (10.5) | 119.2 (12.7) | 116.6 (12.1) | <0.001 |
| **Unmatched Sample** e**xcluding NICU cohort** | |  |  |  |  |
| **N (%)** | **23 (12.0)** | **81 (42.2)** | **60 (31.3)** | **28 (14.6)** | ***P-*value** |
| DCCS | 74.5 (15.3) | 85.4 (13.5) | 110.9 (14.6) | 94.5 (17.5) | <0.001 |
| Flanker | 72.6 (10.9) | 79.7 (9.8) | 99.1 (12.0) | 85.2 (10.5) | <0.001 |
| LSWM | 69.8 (10.1) | 97.8 (12.2) | 96.9 (12.0) | 113.6 (12.7) | <0.001 |
| PCPS | 84.7 (18.9) | 82.9 (17.4) | 115.9 (18.1) | 92.0 (16.0) | <0.001 |
| PSM | 82.3 (10.4) | 98.9 (15.2) | 99.1 (18.0) | 117.9 (14.5) | <0.001 |
| ORT | 79.5 (9.6) | 94.6 (10.9) | 99.8 (12.8) | 123.2 (15.4) | <0.001 |
| PV | 79.7 (11.8) | 95.3 (9.2) | 98.0 (11.2) | 118.9 (13.0) | <0.001 |
| Fluid Composite Index | 62.9 (11.5) | 82.0 (10.1) | 106.7 (14.8) | 100.8 (11.4) | <0.001 |
| Crystalized Composite Index | 76.3 (9.4) | 94.1 (9.5) | 98.8 (11.8) | 124.6 (15.2) | <0.001 |

One-way analysis of variance (ANOVA) with Holm-Bonferroni correction for multiple testing.
DCCS, Dimensional Card Change Sort; LSWM, List-Sorting Working Memory; NICU, neonatal intensive care unit; ORT, Oral Reading Test; PCPS, Pattern Comparison Processing Speed; PSM, Picture Sequence Memory; PV, Picture Vocabulary.
